# Porcine Reproductive and Respiratory Syndrome Virus, Thailand, 2010–2011

**DOI:** 10.3201/eid1812.111105

**Published:** 2012-12

**Authors:** Dachrit Nilubol, Thitima Tripipat, Tawatchai Hoonsuwan, Khampee Kortheerakul

**Affiliations:** Author affiliation: Chulalongkorn University, Bangkok, Thailand

**Keywords:** porcine reproductive and respiratory syndrome virus, nonstructural protein 2, Thailand, Arteriviridae, viruses, pigs, porcine reproductive and respiratory syndrome, PRRS, PRRSV

## Abstract

Characterization of porcine reproductive and respiratory syndrome virus (PRRSV) isolates from pigs in Thailand showed 30-aa discontinuous deletions in nonstructural protein 2, identical to sequences for highly pathogenic PRRSV. The novel virus is genetically related to PRRSV from China and may have spread to Thailand through illegal transport of infectious animals from bordering countries.

Porcine reproductive and respiratory syndrome (PRRS) has a substantial economic effect on the swine industry worldwide. PRRS virus (PRRSV), a member of the family *Arteriviridae*, is the etiologic agent of the syndrome. PRRSVs are divided into 2 distinct genotypes: type 1 and type 2. The genotypes have a similar genomic organization, and 10 open reading frames (ORFs) have been identified (*1*–*3*). Nonstructural protein 2 (Nsp2) and ORF5 are the most variable regions (*4*,*5*), coding for replicase protein and neutralizing epitope, respectively.

In general, PRRSV causes a disease characterized by reproductive failure in sows and respiratory infection in growing pigs. However, in June 2006, a disease characterized by high fever and associated with a high mortality rate emerged in the People’s Republic of China (PRC), resulting in the death of >20 million pigs (*6*). The disease, referred to as porcine high fever disease (PHFD), was caused by a new PRRSV variant with a unique hallmark: 2 discontinuous 30-aa deletions in Nsp2. The variant, identified as a highly pathogenic (HP) PRRSV, has subsequently become endemic in PRC (*7*), and it has spread to other countries, including Vietnam (*8*) and Lao People’s Democratic Republic (Lao PDR) (*9*).

It is thought that HP-PRRSV spread to Thailand early in 2010. Pigs on a small farm in Nong Khai, a border province in northeastern Thailand located near Lao PDR, showed signs of illness identical to those for PHFD. Within 2 weeks of the initial outbreak, similar clinical features were observed in pigs on 19 small farms in a nearby village. Since then, pigs exhibiting similar clinical signs have been observed in >100 herds in >20 provinces throughout Thailand. The causative agent was isolated from sick pigs and determined to be PRRSV. 

To further our knowledge about PRRSV in Thailand, we genetically characterized partial Nsp2 and complete ORF5 genes of PRRSV isolates. In addition, we determined sickness and mortality rates on affected farms.

## The Study

During August 2010–June 2011, outbreaks of disease consistent with PHFD were investigated on 4 pig farms located in geographically separate regions of Thailand (Table 1). Herds were selected for study if farm owners agreed to participate. Pigs in all 4 herds had similar clinical signs. In 3 herds, the outbreak was initially observed in the breeding herd and lasted for ≈1 month; most deaths occurred in the third week. In those 3 herds, the initial signs of illness in sows were inappetence and high fever (40°C–42°C), followed by reddened skin and abortion. Illness rates among sows were 100%, 50%, and 60%, respectively for the 3 herds. The highest number of deaths among the sows occurred within 1 week of onset of the first symptoms. The percentage of culled sows on the 3 farms was 20.4%, 13.6%, and 6.7%, respectively; abortion rates were 52.8%, 8.4%, and 8.7%, respectively (Table 1). The outbreak in the fourth herd was confined to nursery facilities housing ≈4,000 pigs; nearly all pigs were sick within 1 week, and the mortality rate approached 60% within 2 weeks. 

**Table 1:** Characteristics of pig farms with herds infected by PRRSV, Thailand, 2010–2011*

Herd ID, geographic location in country	Production system	Herd size, no. sows	Used attenuated North American PRRSV vaccine	No. (%) sow losses†		No. (%) sows that aborted
				Died	Culled	
UDT, northeast	Farrow-to-wean	500	No	48 (9.6)	102 (20.4)	264 (52.8)
UD, north	Farrow-to-wean	1,500	NK	ND	ND	ND
SCP, west	Farrow-to-finish	500	Yes	153 (30.6)	68 (13.6)	42 (8.4)
FDT, central	Farrow-to-finish	1,200	Yes	29 (2.4)	80 (6.7)	104 (8.7)

*PRRSV, porcine reproductive and respiratory syndrome virus; ID, identification; NK, not known; ND, no data available. †Data are for the 4 weeks following the start of the outbreak on each farm.

We performed PCR on serum samples from sick pigs to determine the presence of PRRSV; previously reported primers (*7*,*10*) were used to amplify partial Nsp2 and complete ORF5 genes. Products were cloned and sequenced at Bio Basic Inc. (Markham, Ontario, Canada). ClustalW (*11*) was used to align nucleotide and deduced amino acid sequences; 18 partial Nsp2 and 58 complete ORF5 genes were analyzed (Table 2).

**Table 2:** PRRSV isolates obtained for sequence analysis from infected pig herds, Thailand, 2010–2011*

Isolate no.	Isolate name	Year and month collected	Genotype	Genes analyzed	GenBank accession no.
1	UD1210EU24/3	2010 Dec	I	ORF5	JX183110
2	UD1210EU23/2	2010 Dec	I	ORF5	JX183111
3	UD1210EU24/1	2010 Dec	I	ORF5	JX183112
4	SCP1210EU7/79-A07	2010 Dec	I	ORF5	JX183113
5	UD1210EU24/2	2010 Dec	I	ORF5	JX183114
6	UD1210EU24/1	2010 Dec	I	ORF5	JX183115
7	UD1210EU25/2	2010 Dec	I	ORF5	JX183116
8	UD1210EU25/1	2010 Dec	I	ORF5	JX183117
9	SCP0311EU1/3	2011 Mar	I	ORF5	JX183118
10	SCP0311EU1/2	2011 Mar	I	ORF5	JX183119
11	FDT0111EU2/3	2011 Mar	I	ORF5	JX183120
12	FDT0111EU2/2	2011 Mar	I	ORF5	JX183121
13	UD1210EU23/3	2010 Dec	I	ORF5	JX183122
14	SCP0311EU1/1	2011 Mar	I	ORF5	JX183123
15	FDT0111EU1/2	2011 Mar	I	ORF5	JX183124
16	SCP0311EU3/1	2011 Mar	I	ORF5	JX183125
17	FDT0111EU1/1	2011 Mar	I	ORF5	JX183126
18	FDT0111EU2/1	2011 Mar	I	ORF5	JX183127
19	SCP0311EU3/2	2011 Mar	I	ORF5	JX183128
20	UDT0810US_5/28–160	2010 Dec	II	ORF5	JN255819
21	UDT0810US_5/28–161	2010 Dec	II	ORF5	JN255820
22	UDT0810US_5/28–162	2010 Dec	II	ORF5	JN255821
23	UDT0810US_5/28–163	2010 Dec	II	ORF5	JN255822
24	UDT0810US_5/28–164	2010 Dec	II	ORF5	JN255823
25	UDT0810US_5/28–165	2010 Dec	II	ORF5	JN255824
26	UDT0810US_5/28–166	2010 Dec	II	ORF5	JN255825
27	UDT0810US_5/28–167	2010 Dec	II	ORF5	JN255826
28	UD1210US/61-E03	2010 Dec	II	ORF5	JN255827
29	UD1210US/61-F03	2010 Dec	II	ORF5	JN255828
30	UD1210US/61-G03	2010 Dec	II	ORF5	JN255829
31	UD1210US/62-H03	2010 Dec	II	ORF5	JN255830
32	UD1210US/62-A04	2010 Dec	II	ORF5	JN255831
33	UD1210US/62-B04	2010 Dec	II	ORF5	JN255832
34	UD1210US-25–1	2010 Dec	II	ORF5	JN255833
35	FDT10US-2–1	2010 Dec	II	ORF5	JN255834
36	FDT10US-2–2	2010 Dec	II	ORF5	JN255835
37	FDT10US-2–3	2010 Dec	II	ORF5	JN255836
38	SCP1210-U.S.-7–79–1	2010 Dec	II	ORF5	JN255837
39	SCP1210-U.S.-7–79–2	2010 Dec	II	ORF5	JN255838
40	UDT0810_E02	2010 Dec	II	Partial Nsp2	JN255839
41	UDT0810_C02	2010 Dec	II	Partial Nsp2	JN255840
42	SCP1210_H02	2010 Dec	II	Partial Nsp2	JN255842
43	SCP1210_B03	2010 Dec	II	Partial Nsp2	JN255841
44	FST0311_C03	2010 Dec	II	Partial Nsp2	JN255843
45	UD1210 (31)14–1	2010 Dec	II	Partial Nsp2	JN255844
46	UD1210 (31)14–2	2010 Dec	II	Partial Nsp2	JN255845
47	1–13(30)UD-1	2010 Dec	II	Partial Nsp2	JN255846
48	UD1210 (31)13–2	2010 Dec	II	Partial Nsp2	JN255847
49	FDT10_3/2	2010 Dec	II	Partial Nsp2	JN255848
50	FDT_3/1	2010 Dec	II	Partial Nsp2	JN255849
51	FDT_2/1	2010 Dec	II	Partial Nsp2	JN255852
52	FDT_2/2	2010 Dec	II	Partial Nsp2	JN255851
53	FDT_2/3	2010 Dec	II	Partial Nsp2	JN255850
54	FST0311_54–4.1	2010 Dec	II	Partial Nsp2	JN255853
55	FST0611_G03	2010 Dec	II	Partial Nsp2	JN255854
56	FST0611_F03	2010 Dec	II	Partial Nsp2	JN255855
57	FST0611_E03	2010 Dec	II	Partial Nsp2	JN255856
58	US65DPI-2	2010 Dec	II	Partial Nsp2	JN255857
*PRRSV, porcine reproductive and respiratory syndrome virus; ORF, open reading frame; Nsp2, nonstructural protein 2.					

**Figure 1:**
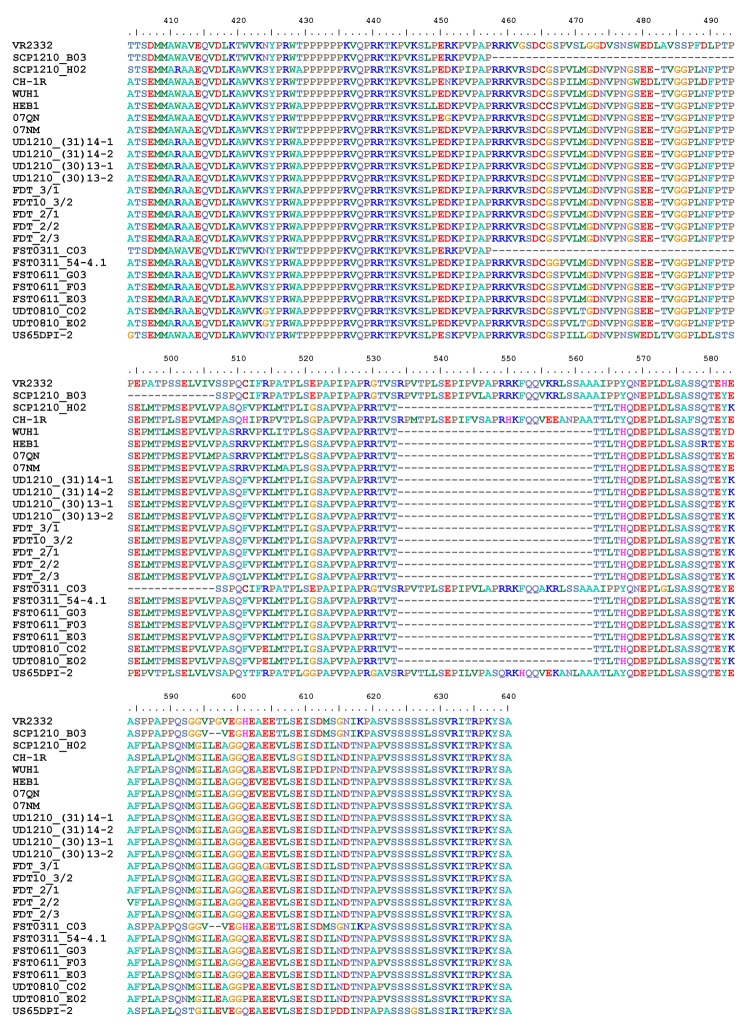
Alignment of amino acid sequences of partial nonstructural protein 2 corresponding to aa 404–640 of ORF1a for porcine reproductive and respiratory syndrome virus (PRRSV) isolates. Sequences are for PRRSV from infected herds in Thailand; highly pathogenic PRRSV isolates from the People’s Republic of China and Vietnam; and strain VR2332, the North American PRRSV prototype. Dashes represent deletions of amino acid residues.

To determine the relationship of PRRSV from herds in Thailand to HP-PRRSV, we compared the partial Nsp2 amino acid sequences corresponding to aa 404–640 of ORF1a from the isolates from Thailand with sequences for HP-PRRSV from PRC and Vietnam and for strain VR2332. PRRSV isolates from Thailand possess 2 discontinuous 30-aa deletions (aa 482 and 534–562) that are identical to those in HP-PRRSV (Figure 1). 

**Figure 2:**
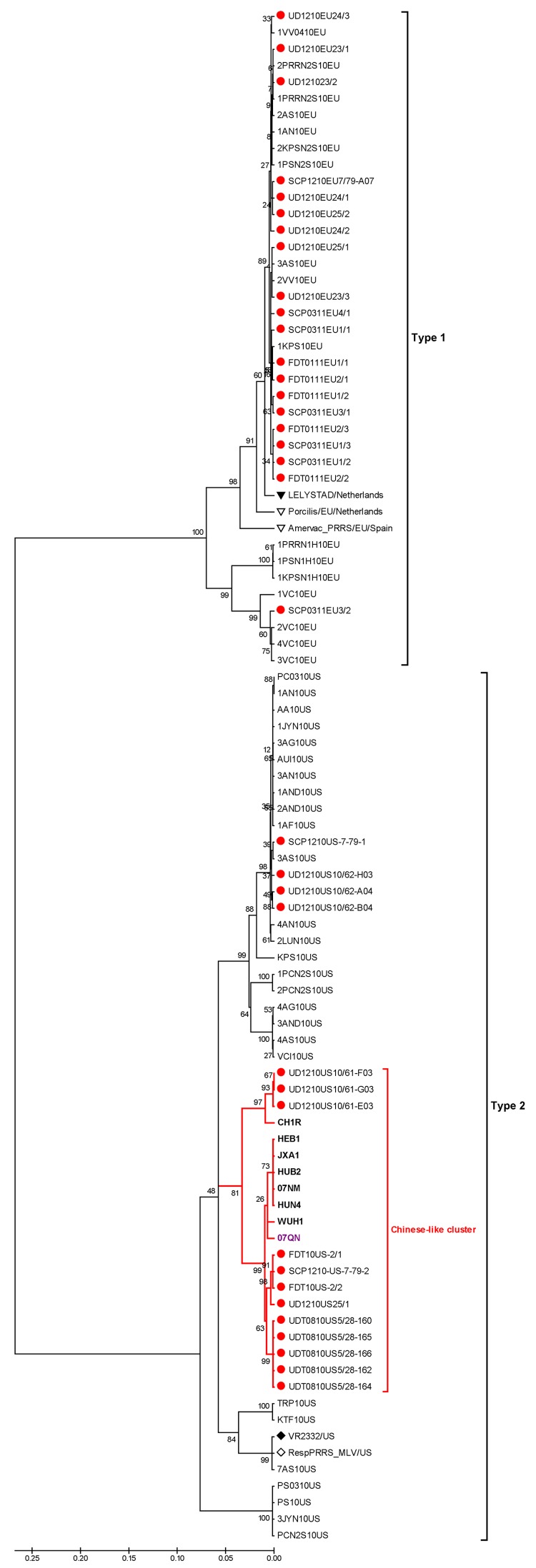
Phylogenetic analysis of types 1 and 2 porcine reproductive and respiratory syndrome virus (PRRSV) isolates constructed by the neighbor-joining method and based on the nucleotide sequences of complete ORF5 genes. The analysis included the following: previous and recent isolates (solid red circles) from herds in Thailand that had an outbreak of HP-PRRSV; European references, including Lelystad virus (solid triangle) and 2 type 1 modified live vaccines (Porcilis PRRS, MSD Animal Health, Boxmeer, the Netherlands; and AMERVAC PRRS, Hipra, Spain) from Europe (open triangles); North American references, including VR2332 (solid diamond) and North American modified live vaccines (Ingelvac PRRS MLV, Boehringer Ingelheim, USA) (open diamonds); modified live vaccines from the People’s Republic of China (CH1R) (open square); isolates from the People’s Republic of China (**boldface**); and isolate from Vietnam (purple font). Scale bar indicate nucleotide substitutions per site; numbers at nodes represent the percentage of 1,000 bootstrap replicates.

To analyze the ORF5 genes of isolates from Thailand, PRC, and Vietnam, we constructed a phylogenetic tree by using the distance-based neighbor-joining method as implemented in MEGA4 (*12*). Bootstrap analysis was performed with 1,000 replicates. The tree showed the co-existence of HP-PRRSV types 1 and 2 in pigs in Thailand (Figure 2). Type 1 isolates from all 4 examined herds clustered with previously reported clusters (*13*,*14*) distinct from type 1 modified live vaccine viruses (Porcillis PRRSV and Amervac PRRS). In contrast, some of the type 2 isolates from affected herds in Thailand had formed a novel cluster distinct from previously reported clusters (*13*,*14*). The novel type 2 isolates from Thailand clustered with isolates from PRC and Vietnam that were associated with PHFD. Genetic similarities between the novel type 2 isolates and HP-PRRSV were 97.8%–98.5% and 96.5%–99.0% homologous at the nucleotide and amino acid levels, respectively. However, the novel type 2 isolates from Thailand were more closely related to the 07QN isolate from Vietnam (98.5% nt and 99.0% aa similarities) than to the isolates from PRC. 

We further investigated routes by which the virus spread. Before the outbreaks in Thailand, dead pigs were illegally transported from Lao PDR to an illegal slaughterhouse located not far from the farm where the first outbreak occurred, and the owner of the farm often visited the slaughterhouse. These findings suggest the movement of infected pigs in neighboring countries might play a role in introducing HP-PRRSV to new regions.

Infected pigs that were transported across the country and illegal slaughterhouses were the most likely routes of the spread of PRRSV within Thailand. The owners of several of the herds we investigated reported that pigs showed clinical signs within 1–2 days after trucks hauling dead pigs arrived at their farms. It was reported that dead pigs from herds in outbreak areas had been loaded on the trucks the day before they arrived at these farms. In Thailand, unlike in the United States, dead pigs are not composted, buried, or incinerated; instead, they are sold to feed catfish. Truckers associated with this trade visit pig farms to buy and transport dead pigs. These trucks are not washed, so they are a potential source of contamination on farms.

Another source for the introduction of the novel PRRSV into Thailand could be an unapproved vaccine from PRC. The phylogenetic tree demonstrated that 3 recent isolates from Thailand (UD1210US/61-F03, UD1210US/61-G03, and UD1210US/61-E03) were more genetically related to CH-1R (an attenuated vaccine strain used in PRC) than HP-PRRSV (Figure 2). CH-1R is a classical PRRSV from PRC that does not possess the 2 discontinuous 30-aa deletions in Nsp2 (*15*). Furthermore, CH-1R is an attenuated PRRSV vaccine strain in PRC, and there is evidence that it has been illegally smuggled into Thailand. Thus, it is possible that this modified live virus from PRC may have been administered to the herd involved in the initial outbreak in Thailand and may have been the source of the novel PRRSV strain that caused the outbreak.

## Conclusions

A novel PRRSV, which is genetically related to PRRSV isolates from PRC, has been introduced into Thailand. Sequences of Nsp2 revealed a unique 30-aa discontinuous deletion in the novel virus, a hallmark of HP-PRRSV. The virus may have been introduced into Thailand through the illegal transport of infected materials from bordering countries, more specifically, from Vietnam to Thailand through Lao PDR. This scenario is supported by our finding that PRRSV isolates from Thailand are more homologous with an isolate from Vietnam than with isolates from PRC. The cause of viral spread within Thailand may have been the movement of infected live and dead pigs across the country.
